# Antidepressant prescribing in the precision medicine era: a prescriber’s primer on pharmacogenetic tools

**DOI:** 10.1186/s12888-017-1230-5

**Published:** 2017-02-08

**Authors:** Chad A. Bousman, Malcolm Forbes, Mahesh Jayaram, Harris Eyre, Charles F. Reynolds, Michael Berk, Malcolm Hopwood, Chee Ng

**Affiliations:** 1 Department of Psychiatry, Melbourne Neuropsychiatry Centre, University of Melbourne, 161 Barry Street, Level 3, Parkville, VIC 3053 Australia; 2 Department of General Practice, The University of Melbourne, Parkville, VIC Australia; 3 Centre for Human Psychopharmacology, Swinburne University of Technology, Hawthorne, VIC Australia; 4 Florey Institute of Neuroscience and Mental Health, The University of Melbourne, Parkville, VIC Australia; 5 Deakin University, IMPACT Strategic Research Centre, School of Medicine, Geelong, Australia; 6 Discipline of Psychiatry, The University of Adelaide, Adelaide, South Australia Australia; 7 University of Pittsburgh School of Medicine, Pittsburgh, USA

**Keywords:** Precision medicine, Pharmacogenomics, Major depressive disorder, Psychiatry, Decision support

## Abstract

About half of people who take antidepressants do not respond and many experience adverse effects. These detrimental outcomes are in part a result of the impact of an individual’s genetic profile on pharmacokinetics and pharmcodynamics. If known and made available to clinicians, this could improve decision-making and antidepressant therapy outcomes. This has spurred the development of numerous pharmacogenetic-based decision support tools. In this article, we provide an overview of pharmacogenetic decision support tools, with particular focus on tools relevant to antidepressants. We briefly describe the evolution and current state of antidepressant pharmacogenetic decision support tools in clinical practice, followed by the evidence-base for their use. Finally, we present a series of considerations for clinicians contemplating use of these tools and discuss the future of antidepressant pharmacogenetic decision support tools.

## Background

Antidepressant use has increased over the past decade [1] but only half of those taking them will respond [2] and about 55% will experience at least one bothersome side effect [3]. In the largest and longest evaluation of antidepressants, the Sequenced Treatment Alternatives to Relieve Depression (STAR*D) trial, it took more than 50 weeks and at least four trials to obtain a cumulative remission rate of 67% [4]. Such suboptimal outcomes as these has resulted in a recent call for better use of antidepressants, including searching for predictors of response and reducing usage in people with situational and personality based problems [5]. Current pharmacological strategies include swifter dose escalation and medication changes as well as augmentation strategies [6]. An emerging and promising strategy is to utilise a person’s pharmacokinetic and pharmacodynamic genetic profile to guide individualised antidepressant therapy decisions. Increasingly evidence indicates that genetic factors play a critical role (42–50%) in determining the differences in both response to and adverse effects of antidepressants [7, 8] and this evidence has in part served as the foundation of precision medicine.

Precision medicine is a novel approach to disease prevention and treatment. It is based on an appreciation of the heterogeneity of disease entities and individual difference in genetic make-up. This approach has had its fair share of criticism, since use of genetics alone can be construed as stigmatising or unaffordable to most. On the other hand, others have argued that this may be a means to better understand issues related to treatment response or lack of due to specific genetic characteristics and could be a useful tool eventually enabling universal access [9]. Pharmacogenetic application to antidepressant prescription aims to both improve remission rates for depression and reduce adverse effects associated with antidepressants by identifying genetic markers that could be utilized as clinical tools for tailoring treatment. Currently, there are a number of pharmacogenetic decision support tools that are commercially available [10] but recent commentary within the field suggests the widespread adoption of these tools in practice may be premature [11–15]. However, pharmacogenetic tools continue to be refined, developed, and marketed to clinicians who have varying degrees of knowledge of the nature and/or the current evidence of these tools. As such, this paper aims to provide an introduction to pharmacogenetic decision support tools relevant to antidepressants and raise a number of considerations for clinicians who may be contemplating use of these tools in their practice.

### The evolution of pharmacogenetic decision support tools

Pharmacogenetic decision support tools have evolved rapidly over the past decade. In 2004, the first-generation of clinical pharmacogenetic tools was born when Roche’s *CYP2D6* and *CYP2C19* Amplichip (Basel, Switzerland) was made available. First-generation tools test individual genes/variants and provide genotype and accompanying phenotype (e.g. metaboliser status) information. However, they do not account for potential synergies between genetic variants and may not offer clinical interpretation/recommendations or flag drug-drug interactions. Although individual gene tests remain available for clinical use, second-generation tools now utilise combinatorial or polygene testing. The combinatorial/polygene approach is based on evidence that most antidepressants and other psychiatric medications interact with multiple pharmacodynamic and pharmacokinetic pathways [16]. Thus, unlike their first-generation predecessors, second-generation tools [see recent reviews: [10, 17] account for synergies between genes included in their testing panels and often provide drug-drug interaction information to aide in drug selection and/or dosing decisions.

**Fig. 1:**
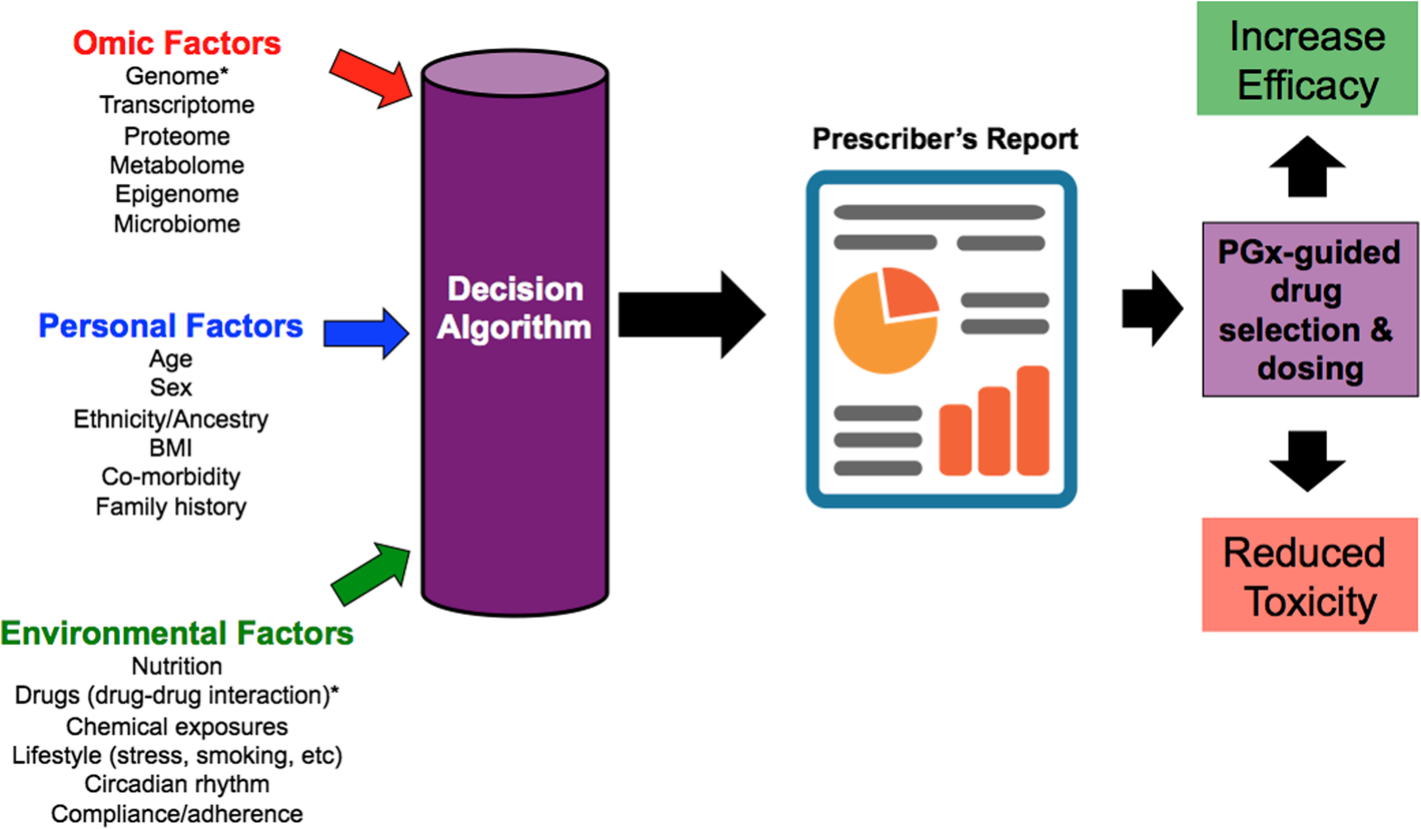
Overview of current and future development of pharmacogenetic-based decision support tools. *These factors are typically included in decision algorithms of currently available pharmacogenetic-based decision support tools

Ideally, pharmacogenetic-based decision support tools would include information from a wide variety of genomic, personal, and environmental factors implicated in drug response and toxicity variability, yet current tools typically only include genetic and sometimes drug-drug interaction information (Fig. 1). This is an issue since many of the most robust predictors of response are clinical and psychosocial in nature [18]. The genetic content varies considerably from tool to tool, although what is consistent across all antidepressant pharmacogenetic tools is a focus on pharmacokinetic genes, specifically *CYP2D6* and *CYP2C19* [10]. The focus on *CYP2D6* and *CYP2C19* is primarily a result of expert groups such as the Clinical Pharmacogenetics Implementation Consortium [19] that have developed dosing guidelines for serotonin selective reuptake inhibitors and tricyclic antidepressants based exclusively on *CYP2D6* and *CYP2C19* genetic variation [20–22]. Despite these guidelines, a majority of tools also include other pharmacokinetic and/or pharmacodynamics genes in their testing panels with varying degrees of evidence [10]. These gene panels are then subjected to the tool’s decision algorithm, which in turn produces an interpretative report. Interpretative reports vary in the depth of content but at minimum include a snapshot of the patient’s pharmacogenetic status along with recommendations and/or considerations aimed at optimising efficacy and/or reducing adverse events associated with antidepressant therapy.

### Current state of pharmacogenetic decision support tools in practice

**Fig. 2:**
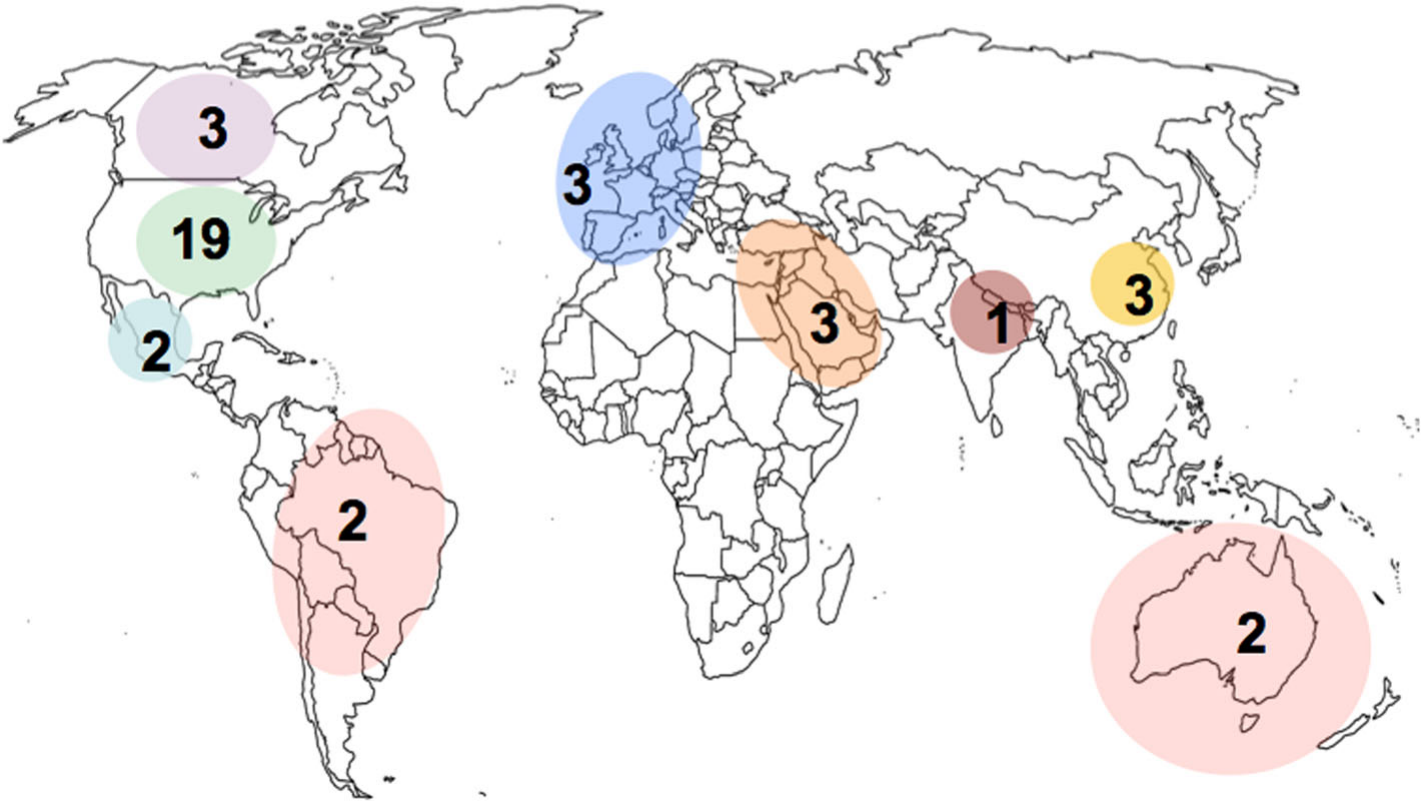
Number of pharmacogenetic-based decision support tools available by country/region

The menu of tools available to clinicians depends on geography (Fig. 2). Clinicians in the United States have the largest selection of tools to choose from, although the availability of tools in other countries and regions of the world is growing. As a result, pharmacogenetic testing for psychotropic medication use is increasing among physicians in the United States [23] and Canada [24]. However, there is less support for direct-to-consumer genetic testing and three-quarters of US psychiatrists believed genetic counselling would be needed for patients having testing [23]. A recent study reported that 6% of psychiatrists in the United States ordered a pharmacogenetic test in the past six months, representing 47% of all genetic tests ordered by psychiatrists [25]. In other parts of the developed world, similar studies have not yet been conducted but there is no reason to think the opinions and rates of genetic testing are likely to differ. However, it remains unclear at what stage of treatment these tests are ordered or what underlying clinical circumstances led to the decision to order a pharmacogenetic test.

Developers of pharmacogenetic tools advocate for pre-emptive (i.e. prior to prescribing) use of their tools but we [10] have argued these tools are more likely to be ordered in a reactive (post-prescribing) fashion. A recent case series has shown the utility of pharmacogenetic testing for a patient that had failed to respond to multiple medications and for another that was experiencing a high side-effect burden [26], limitations of this evidence type notwithstanding, and that other mechanisms such as the nocebo effect may be operative [27]. As such, the evidence base for both pre-emptive and reactive use of pharmacogenetic tools for antidepressant therapy remains limited. Furthermore, there is debate around whether pharmacogenetic tools will boost antidepressant treatment adherence, with some arguing pharmacogenetic tools may assist in doctor-patient shared decision making, improve health literacy, reduce the perception of side effects and poor efficacy or treatment, and reduce health care costs [28]. In fact, a recent retrospective study of claims data implied an increase in adherence among patients for whom a polygene pharmacogenetic tool was ordered compared to those who received standard treatment [29]. Although prospective trials will be need to confirm these findings, promoting adherence is clearly desirable given data suggesting 42% of patients discontinue antidepressant treatment after 12 weeks [30] and long-term adherence is estimated to be 45% [31].

### Evidence-base for pharmacogenetic decision support tools

Three recent reviews have assessed the evidence-base of pharmacogenetic-guided antidepressant prescribing using different evaluation frameworks [10, 17, 32]. All reviews highlight that only a small proportion (<20%) of current pharmacogenetic tools have been empirically evaluated and suggest that pharmacogenetics has potential for clinical utility but there are numerous gaps in the current evidence. For example, it is unclear whether these pharmacogenetic-guided tools can shorten the time to remission and/or sustain the duration of remission from depression. Furthermore, the utility of these tools for assisting in the decision to switch or augment a patient’s current prescription remains uncertain, although preliminary evidence suggests these tools may assist with medication changes [33]. Finally, the cost-effectiveness of pharmacogenetic decision support tools remains unclear [34] and until robust economic studies are conduced, firm clinical guidelines or recommendations cannot be made.

### Considerations about the use of pharmacogenetic decision support tools

There are a number of factors to consider if and when selecting a pharmacogenetic decision support tool and a variety of evaluation frameworks are available to clinicians to guide this process. One of the most well-known is the Oxford Centre for Evidence-Based Medicine (CEBM) Levels of Evidence [35]. The CEBM Levels of Evidence assists clinicians in identifying and appraising evidence using a hierarchy of the likely best evidence. In this hierarchy, systematic reviews of RCTs and individual RCTs are the preferred sources of evidence for making appraisals about a particular intervention. As mentioned above, three systematic reviews of these tools for antidepressant therapy have been completed [10, 17, 32] and two individual RCTs have been conducted [36, 37]. The CEBM advises that even in cases where supportive evidence exists, clinicians should consider at minimum four questions before choosing to use or adopt the intervention. Below we present these questions and provide salient information to assist clinicians in forming their own conclusions.

1. *Do you have good reason to believe that your patients are sufficiently similar to the patients in the studies*? It has previously been noted that the studies conducted to date have primarily included Caucasian females in their forties who lacked common comorbidities (e.g. substance use disorder) among patients with depression [17]. Although the over-representation of females is common in depression clinical trials (and clinical practice) and may limit the application in real world clinical settings, the under-representation of non-Caucasian patients is particularly noteworthy given the multi-ethnic populations of most developed countries.

Ethnicity and its accompanying cultural and environmental factors account for some inter-individual pharmacokinetic and pharmacodynamic genetic variation relevant to antidepressant therapy [38]. For example, the alleles used to predict *CYP2D6* and *CYP2C19* metaboliser status vary considerably in frequency between ethnicities [39–41] and tools including *CYP2D6* and *CYP2C19* do not necessarily measure the same alleles. This is a particularly important issue when contemplating whether to order a pharmacogenetic test for a non-Caucasian patient, in that most of the tools were developed and tested in Caucasian populations and may not include alleles that are rare in in this population but more frequent in people of Asian and/or African descent. As a result, non-Caucasians may be reported as normal (i.e. extensive) metabolisers by default when in fact they are poor or ultra-rapid metabolisers. Thus, it is important to be aware that ‘predicted’ metaboliser status provided by all pharmacogenetic tools should always be interpreted in the context of diverse cultural and environmental factors. In fact, a recent comparison of genotype-predicted and ‘true’ *CYP2D6* metabolism showed genotype-predicted metaboliser status missed 43% to 64% of true poor metabolisers, depending on ethnicity [42]. It should also be noted that within the broad Caucasian, Asian, and African ethnic groupings, allelic differences in key pharmacogenes (e.g. *CYP2D6* and *CYP2C19*) have been observed, although more subtle than difference observed between these broad groupings [43–45]. Such ethnic and cultural variations need to be addressed in future pharmacogenetic tool development.

2) *Does the tool have a clinically relevant benefit that outweighs the harms*? Evidence to date suggests there may be potential benefits associated with the use of pharmacogenetic decision support tools, such as increased remission rates [36, 46–48], reduced adverse effects [36, 49], and cost-savings [36, 50–52]. However, the clinical utility of these tools remains uncertain due to a lack of high quality randomized clinical trials with adequate statistical power. Nonetheless, the prevailing opinion is that antidepressant pharmacogenetic testing is of relatively low risk. Exceptions arise when pre-emptive testing results may take a significant period (range: one day – three weeks) to be available. Delaying initiation of antidepressant therapy whilst awaiting results of the test may not be ethically appropriate and may lead to clinical deterioration. These potential harms could be mitigated via the deployment of point-of-care testing. Unfortunately, none of the antidepressant pharmacogenetic tools offer point-of-care testing but examples from antiplatelet prescribing [53] suggest such testing is likely to become feasible and applicable to antidepressant prescribing.

Another potential risk is loss of genetic privacy. Although privacy concerns are not unique to pharmacogenetic testing, it has been argued that genetic data is perceived as being higher quality and more definitive than other laboratory data, suggesting special protections are warranted [54]. However, not all genetic data is equal. Most antidepressant pharmacogenetic tools do not included genetic variants that are used to identify risk or diagnosis of disease and as such likely do not require additional privacy measures beyond those in place for other laboratory and clinical data. However, we are aware of pharmacogenetic tools that measure genetic variation in apolipoprotein E (*APOE*), a gene with potential risk implications for Alzheimer disease, as well as emerging tools that will employ genome and exome sequencing technology that have the capability of identifying disease-related mutations. Thus, mitigation of perceived and real genetic privacy concerns will continually need to be addressed by regulators, developers, and end-users of pharmacogenetic tools. To date, these issues have been addressed in some countries via genetic discrimination legislation such as the US Genetic Information Nondiscrimination Act of 2008 [55]. Most pharmacogenetic companies and laboratories offering pharmacogenetic testing use encrypted emails and password-protected websites to gain access to genetic information. However, the potential for this information to be inadvertently shared outside of the patient-clinician relationship is not trivial and for some patients could reduce their enthusiasm to be tested [56].

3) *Is another tool better?* To our knowledge, no comparative effectiveness trial of pharmacogenetic decision support tools has been conducted. However, given that multiple tools are available for use worldwide, such a trial is a priority and would ideally be funded and conducted independent of the tool developers to avoid potential biases.
4) *Are the patient’s values and circumstances compatible with use of pharmacogenetic decision support tools?* Such consideration will obviously vary from patient to patient and may be influenced by cultural, spiritual, and/or historical factors. A recent U.S. national survey of public attitudes toward pharmacogenetic testing suggested the majority (>73%) of people are interested in pharmacogenetic testing to assist with drug selection, guide dosing and/or predict side effects. A survey of 910 undergraduate medicine and science students showed 90% were in favor of pharmacogenetic testing [57]. Furthermore, a telephone survey of US adults reported younger Caucasians with a college education and history of side effects from medication were more likely to be interested in testing but most (73%) respondents’ would not agree to pharmacogenetic testing if there was a risk that their genetic material or information would be shared without their permission [56].

## Conclusion

In the precision medicine era, the supply and demand for antidepressant and other drug pharmacogenetic testing is anticipated to increase. With this in mind, it is feasible that clinicians will soon be able to obtain genetic information and generate a report that provides personalised treatment recommendations within a single consultation. Given the potential to improve patient treatment outcomes, even a modest increase in remission rates of depression or reduction of adverse event risk would significantly reduce the growing disease burden of depression at the population level. However, the availability of pharmacogenetic testing and genetic information to clinicians does not guarantee clinical applicability. More independent research related to the effectiveness and utility of pharmacogenetic tools in real world practice is needed, particularly within the primary care setting, where the majority of patients with depression are diagnosed and treated [58]. In the next five years, results will be available from a number of randomized clinical trials currently underway in the U.S and Canada that will allow for a better evaluation of the clinical utility of antidepressant pharmacogenetic decision support tools. In the meantime, new tools will continue to emerge and diffuse into practice. As such, clinicians are encouraged to consider the evidence-base of these tools in the context of their practice and their diverse patient needs.
